# Abdominal Epilepsy

**Published:** 1906-08-18

**Authors:** 


### Progress in Medicine and Surgery.

### ABDOMINAL EPILEPSY.

With the occurrence of abdominal symptoms in epilepsy we are sufficiently familiar. Among the commonest of the phenomena which constitute an *aura* and usher in an attack of convulsions are abnormal sensations in the epigastrium or intestine, feelings of uneasiness or distress, heartburn or palpitation. Familiar also is the fact that gastro-intestinal disturbance often appears to precipitate an outburst of epilepsy. Thus, the presence of intestinal worms, the consumption of an indigestible article of diet, too large a meal, or lateness and irregularity of meals all predispose to the onset of "fits" in susceptible individuals. Occasionally, too, epileptics exhibit voracious appetities, and, unless checked and treated, will consume very large quantities of food. But some cases recently described by Dr. J. W. Russell, in which paroxysmal abdominal pain was noted in association with epilepsy, must be placed in a different category. In these the abdominal symptoms did not simply act as the immediately exciting cause or constitute the *aura,* but they occurred in conjunction with, or, in some cases, replaced, in whole or in part, the ordinary convulsive phenomena. Such pain may be regarded either as an integral part of a genuine epileptic attack, or as due, together with the convulsive symptoms, to a pathological cause in the abdomen acting on a predisposed nervous system. Perhaps not a few cases belong to the former class. Sometimes these abdominal symptoms recur at intervals before major fits have developed, and sometimes, as in a considerable number of cases collected by Herpin, for months, or even years, they replace the fits. Occasionally slight muscular contractions or traces of mental disorder accompany the pain; at other times spasmodic colicky pain, cramp, or tenesmus may be the only thing observed. An associated feeling of fatigue is of decided significance. To give one or two instances, Herpin describes the case of a little French girl, aged nine, who frequently suffered from major fits. In these always the first thing observed was that the child cried out she had a pain in her stomach, and crossed her hands over that region. Her face then became red, her eyes were turned upwards, and she fell in typical clonic convulsions. Sometimes, however, the pain occurred alone; the child cried out as before, and placed her hands on the abdomen, but recovered without change of colour or sign of convulsion. One of Dr. Russell's own cases, a child of six, was brought for treatment for "fainting bouts." The sequence of events was a sudden complaint of feeling "so tired," pain in the stomach lasting for a minute or so, swallowing as though to get rid of something in the throat, sweating and pallor, followed by drowsines and slumber. At the time of observation this little girl had never fallen or shown muscular twitchings, but in another child sharp abdominal pain was followed by decided twitching on both sides of the face. Other cases might be quoted, as that of a gentleman subject to occasional sudden attacks of deep-seated pelvic pain, accompanied by loss of consciousness, rigidity, change of colour, and drowsiness, the whole attack being of brief duration. The diagnosis of abdominal pain as "epileptic" in nature seems to depend on its sudden occurrence, brief duration, and uniform character, on the absence of any abdominal condition which could account for it, and on its association, at one time or another, with the more typical symptoms of an epileptic fit. As has been already mentioned, Russell lays stress on the value of feelings of fatigue as being very characteristic of epileptic attacks. Such sensations may precede the fit or abdominal crisis, or either may be followed by more or less prolonged somnolence. The recognition of the true nature of certain forms of sudden and brief abdominal pain is of decided value from a practical standpoint, as such pain, though it may readily yield to treatment suitable for epilepsy, would prove intractable if treated on purely abdominal lines. The question is now being asked with increasing frequency,

**What are Suitable Remedies** for what the ancients used to call the "sacred disease," and which a recent writer has described as "the strangest disease in human history"—strange in its universal incidence, in its sudden dramatic onset, its often-times tragic conclusions, and in its power in a moment, for the time being, to change the face of existence for the sufferer. Hitherto, although other drugs are employed, the sheet anchor in the treatment of this complaint has been the administration of the potassium or other salt of bromine. The use of the bromides is in various quarters, however, becoming discredited by those who have given much study to epilepsy. But attractive as are the schemes, projected and realised, for the establishment of epileptic colonies and the rational and hygienic individual treatment of the sufferers, and desirable as it is that such colonies should be multiplied and extended, it is probable that for many a long year the majority of the victims of epilepsy must necessarily continue to be treated in the oftentimes unsuitable surroundings of their own homes. Although much may be done by intelligent effort to improve the surroundings and so to diminish the number and severity of attacks, the administration of drugs must still play a large part in treatment. Whilst the bromides do not cure in the true sense, they certainly have a marked effect in diminishing the number of convulsions. At the same time, they have decidedly unpleasant physical effects, and their too free administration may in some cases account for the progressive mental impairment observable in many epileptics. Hence

**A New Remedy** which has a sedative effect without unpleasant and dangerous sequelæ would be much welcomed. *Veratrum viride,* the root of the green hellebore, is claimed to be such a useful sedative, and to be finding favour as such in America and in Italy. A recent writer claims for it decided soothing and calming effects without any tendency to produce cardiac failure if given cautiously and in small initial doses, such as two drops of the tincture every few hours. Larger doses have been given with safety, as in a case of puerperal eclampsia, in which twenty drops were administered every two hours. Veratrum viride is not a new drug. Not long ago it had a place in the British Pharmacopœia, but it was displaced at the last revision. Its action is very complex, and it is so powerful a poison as to be regarded by many as unfit for use. It causes profound cardiac and respiratory depression, vomiting, and intense muscular weakness. The last-named effect appears to be due to a paralysing action on the anterior cornual cells of the spinal cord, a result which offers an explanation of its value as a sedative in epileptic and eclamptic seizures. At the same time, its powerful action in reducing the force and frequency of the pulse indicates its special suitability in convulsive states attended by a high tension pulse and a strongly acting heart, and it has been found useful in some cases of cerebral apoplexy. If its efficiency combined with safety can be proved, veratrum viride will be a welcome addition to the therapeutics of epilepsy, but it must never be forgotten that its violent toxic properties call for the utmost caution and watchfulness when prescribing it.

